# Hemolysis during open heart surgery in patients with hereditary spherocytosis — systematic review of the literature and case study

**DOI:** 10.1186/s13741-024-00411-w

**Published:** 2024-06-10

**Authors:** Konrad Mendrala, Tomasz Czober, Tomasz Darocha, Damian Hudziak, Paweł Podsiadło, Sylweriusz Kosiński, Bogusz Jagoda, Radosław Gocoł

**Affiliations:** 1 Department of Anaesthesiology and Intensive Care, Medical University of Silesia, Katowice, Poland; 2 Department of Cardiac Surgery, Upper-Silesian Medical Centre, Medical University of Silesia, Katowice, Poland; 3 Department of Emergency Medicine, Jan Kochanowski University, Kielce, Poland; 4 Department of Intensive Interdisciplinary Therapy, Jagiellonian University Collegium Medicum, Krakow, Poland

**Keywords:** Hereditary spherocytosis, Hemolysis, Cardiac surgery, Cardiothoracic surgery, CPB

## Abstract

**Background:**

Due to the distinctive nature of cardiac surgery, patients suffering from hereditary spherocytosis (HS) are potentially at a high risk of perioperative complications resulting from hemolysis. Despite being the most prevalent cause of hereditary chronic hemolysis, the standards of surgical management are based solely on expert opinion.

**Objective:**

We analyze the risk of hemolysis in HS patients after cardiac surgery based on a systematic review of the literature. We also describe a case of a patient with hereditary spherocytosis who underwent aortic valve repair.

**Methods:**

This systematic review was registered in the PROSPERO international prospective register of systematic reviews (CRD42023417666) and included records from Embase, MEDLINE, Web of Science, and Google Scholar databases. The case study investigates a 38-year-old patient who underwent surgery for an aortic valve defect in mid-2022.

**Results:**

Of the 787 search results, 21 studies describing 23 cases of HS undergoing cardiac surgery were included in the final analysis. Hemolysis was diagnosed in five patients (one coronary artery bypass graft surgery, two aortic valve bioprosthesis, one ventricular septal defect closure, and one mitral valve plasty). None of the patients died in the perioperative period. Also, no significant clinical hemolysis was observed in our patient during the perioperative period.

**Conclusions:**

The literature data show that hemolysis is not common in patients with HS undergoing various cardiac surgery techniques. The typical management of a patient with mild/moderate HS does not appear to increase the risk of significant clinical hemolysis. Commonly accepted beliefs about factors inducing hemolysis during cardiac surgery may not be fully justified and require further investigation.

## Background

Hereditary spherocytosis (HS) is an inherited genetic disease causing the formation of fragile red blood cells with an abnormal morphology. Defects in essential erythrocyte membrane proteins, such as ankyrin, band 3, or spectrin, are responsible for the loss of structural integrity. This alters their typically biconcave form, which can result in the weakening of the cell structure, rendering them more susceptible to mechanical damage. HS affects around 1 in every 2000 people in the United States and Europe (Perrotta et al. 2008). Individuals with this condition may have a different clinical presentation, but their life expectancy is the same as the general population. Its manifestations include subclinical, mild hemolytic anemia, and severe hemolytic breakthroughs.

Despite being the most prevalent cause of hereditary chronic hemolysis, the existing literature only provides single cases of hereditary spherocytosis in patients undergoing cardiac surgery. Because the disease is uncommon, guidelines for patient management in perioperative care are based on expert opinion, single reports from cardiac surgery, and experience from noncardiac procedures. The pathophysiology of hemolysis in HS is multifactorial and involves several mechanisms. In addition to susceptibility to mechanical stress, factors predisposing to red blood cells (RBC) damage may involve perioperative care, pharmacotherapy, and technical aspects of the surgical procedure. We analyze the risk of hemolysis in HS patients after cardiac surgery based on a systematic review of the literature. We also describe a rare case of a patient with hereditary spherocytosis who underwent aortic valve repair.

## Methods

### Study design and participants

**Fig. 1:**
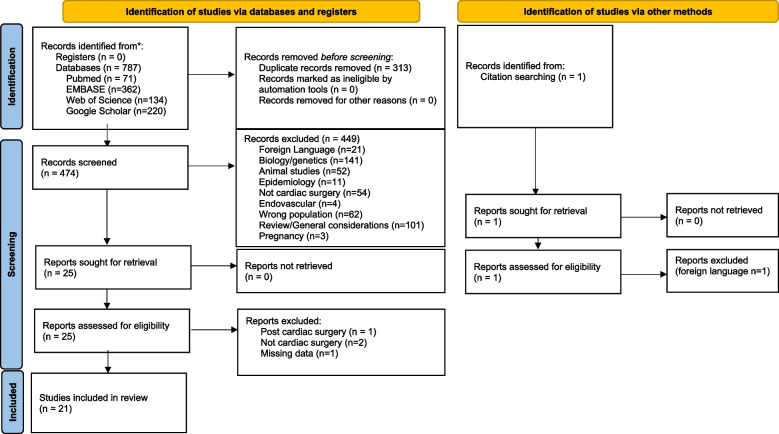
PRISMA flow diagram

This systematic review was registered in the PROSPERO international prospective register of systematic reviews (CRD42023417666). The search included only articles in English with no time limit, up until 30 June 2023. For the analysis of the search results, the web app Rayyan.ai was used (Ouzzani et al. 2016). The results were independently assessed for their relevance by two authors (K. M. and T. D.) according to the predefined criteria: only articles including HS patients undergoing cardiac surgery. Reviews, animal studies, and conference abstracts were not analyzed. In addition, the references of the included studies were searched manually to find any additional studies. Any disagreements were resolved by team discussion. Two review authors (K. M. and T. Cz.) independently extracted data for each included study. PRISMA flowchart is presented in Fig. 1.

### Information sources and search methods

With the help of a professional librarian, we searched Embase, MEDLLINE, Web of Science, and Google Scholar databases using the following MeSH and keywords: “Spherocytosis, Hereditary” [Mesh], “Thoracic Surgery” [Mesh], “Cardiac Surgical Procedures” [Mesh], “Extracorporeal Circulation”, Hereditary Spherocytosis, Surgery, Operation, Procedure, Cardiac, Heart, Myocardial, Cardiomyoplasty, Pericardial, Pericardiectomy, Coronary, Arterial, Bypass, Angioplasty, Revascularization, Valve, Mitral, Tricuspid, Aortic, Transplant, Transplantation, Cardiopulmonary bypass, and Heart–lung.

### Synthesis of results

The main outcome we studied was hemolysis during cardiac surgery in HS patients. Details about type of surgery, technical details of cardiopulmonary bypass (CPB), blood transfusions, and perioperative complications were also recorded. The quality of the publications included in the systematic review was assessed using the tool introduced by Murad et al. and presented in Table 1 (Murad et al. 2018).

**Table 1:**
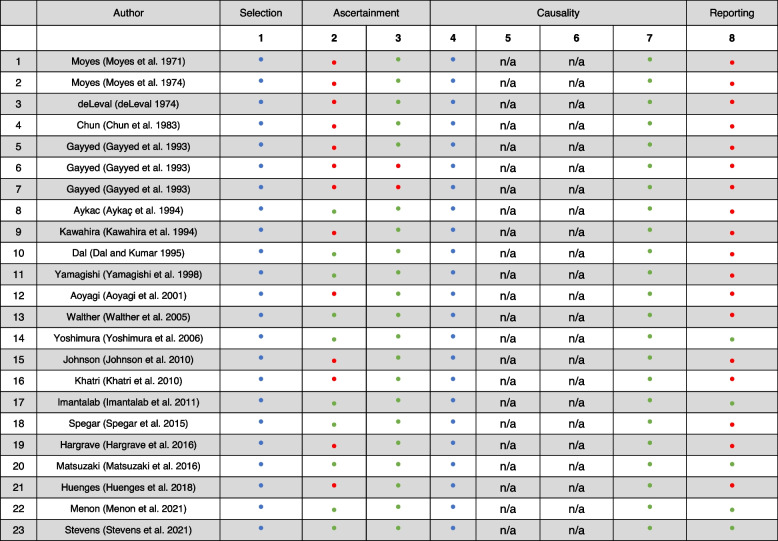
Quality assessment of included case reports and case series (Aykaç et al. 1994; Dal and Kumar 1995; Huenges et al. 2018; Johnson et al. 2010; Khatri et al. 2010; Menon et al. 2021; Moyes et al. 1974; Spegar et al. 2015; Stevens et al. 2021; Walther et al. 2005)

^1^Does the patient(s) represent(s) the whole experience of the investigator (center), or is the selection method unclear to the extent that other patients with similar presentation may not have been reported? ^2^Was the exposure adequately ascertained? ^3^Was the outcome adequately ascertained? ^4^Were other alternative causes that may explain the observation ruled out? ^5^Was there a challenge/rechallenge phenomenon? ^6^Was there a dose-response effect? ^7^Was follow-up long enough for outcomes to occur? ^8^Is the case(s) described with sufficient details to allow other investigators to replicate the research or to allow practitioners make inferences related to their own practice?  Yes.  No.  Not specified

## Results

Of the 787 search results, 21 studies describing 23 cases of HS undergoing cardiac surgery were included in the final analysis. The articles reported on patients between 6 weeks and 69 years of age. Four patients underwent CABG surgery (including one OPCAB), seven patients had valve replacement, five valve plasty, in eight patients ASD/VSD was closed, one patient had HVAD implantation, and one heart transplant. Hemolysis was reported in five patients (21.7%): one CABG, two AVR bioprosthesis, one VSD, and one MVpl. No patient with a mechanical valve was diagnosed with hemolysis. Hemolysis on roller pump (including occlusive pump) and bubble oxygenator was not reported. Hypothermia was implemented in 10 patients, and 3 of these patients with temperature 28, 32, and 34.8 °C were diagnosed with hemolysis. None of the patients died in the perioperative period. Details of the type of surgery and technical aspects are given in Table 2. Laboratory results of the analyzed population are shown in Table 3.

**Table 2:** Patient characteristics and technical aspects of surgery

	**Author**	**Year**	**Age**	**Gender**	**Past medical history**	**Splene-ctomy**	**Surgery**	**CPB**	**CPB priming**	**Temperature °C**	**Cardioplegia**	**Xclamp (min)**	**CPB (min)**	**Total blood loss (hospital)**	**Blood**	**Additional treatment**	**LOS**	**Complicatons**	**Hemolysis**
1	Moyes	1971	16	F	-	Yes	MVpl	Short occusive	Ringer’s solution	-	-	-	-	-	-	-	21	No	No
2	Moyes	1974	5	M	Fallot’s tetralogy	No	VSD clousure, RV infundibiulectomy	Temptrol oxygenator	Ringer’s solution, donors blood	-	-	-	-	-	-	-	20	-	Yes
3	deLeval	1974	-	F	-	Yes	MVR, TVR (mech)	-	-	-	-	-	100	-	-	-	-	No	No
4	Chun	1982	38	F	-	Yes	MVR (mech 25 mm) TVR (mech 29 mm) AVR (mech 21 mm)	-	-	-	-	59	189	-	-	-	-	-	No
5	Gayyed	1993	67	M	HSM	No	AVR (bio 25 mm)	-	-	28	-	-	68	1050	RBC	-	-	Hemolysis	Yes
6	Gayyed	1993	60	F	HF, HSM	No	MVR (bio 33 mm)	-	Packed RBCs	24	-	-	80	400	RBC	-	14	No	-
7	Gayyed	1993	64	M	IHD	Ye	VSD clousure	-	-	24	-	-	110	1950	RBC	-	21	Total parenteral nutrition, renal failure	-
8	Aykac	1994	15	F	-	Yes	MVpl	Roller pump, bubble oxygenator	Total exchange transfusion	Hypotheria	Cold, crystalloid	-	-	-	RBC	-	-	-	No
9	Kawahira	1994	15 months	M	Chomosome 8 defect	No	PDA closure, MVpl, LA plication	-	Poloxamer 188	-	-	55	180	127	RBC	Haptoglobin	-	Hematuria	Yes
10	Dal	1995	31	F	-	Yes	ASD clousure	Harvey 1700 model bubble oxygenator	Plasma, priming fluid, mannitol	34	-	7	35	Minimal	-	-	10	No	No
11	Yamagishi	1998	50	M	-	No	AVR (mech 23 mm)	-	Crystalloid solution	-	-	-	-	800	RBC, FFP (autologus)	Cell saver, ferrous supplementation, rHuEPO	-	No	No
12	Aoyagi	2001	9	F	HSM	Yes	ASD closure	-	Ringer’s solution, mannitol, albumins, hydroxyethylated starch	Normothermic	-	-	30	-	RBC (autologus)	Haptoglobin	14	-	-
13	Walther	2005	6 weeks	-	CM	No	OPCAB	OPCAB	-	-	-	-	-	-	-	-	15	No	-
14	Yoshimura	2006	18 months	F	-	No	VSD closure, PV commissurotomy, PT patch angioplasty	Roller pump	Packed RBCs, albumin	-	-	-	150	-	RBC	Haptoglobin, dilutional ultrafiltration	15	No	No
15	Johnson	2010	6	M	DCM	No	HTX	-	Exchange transfusion of packed RBCs, FFP	-	-	-	-	-	RBC, FFP	Exchange transfusion, aprotinin, ultrafiltration	> 27	Transient neurological complications, CNS hematomas	No
16	Khatri	2010	61	M	IHD, HT, DL	Yes	CABG	-	-	Mild hypothermia, topical ice	Cold, antegrade and retrograde	-	105	-	NO	-	4	No	No
17	Imantalab	2011	60	M	-	Yes	CABG	Medtronic affinity	-	32, topical ice	Cold, antegrade	48	135	-	RBC	Furosemide, NaHCO3, mannitol, ultrafiltration	8	Hematuria, IABP	Yes
18	Spegar	2015	69	M	HT, DL, CVA, AS	Yes	CABG	Roller pump Non-coated, Quadrox oxygenator	Ringer’s solution, mannitol	Normothermia	Cold, antegrade	-	60	-	RBC (autologus)	-	7	No	No
19	Hargrave	2015	28	F	-	No	VSD closure, myectomy, AVR (bio 21 mm), ascending aortoplasty	-	Retrograde autologous priming	34.8–36.6	Normothermic	50	60	-	RBC	Cell saver	7	-	Yes
20	Matsuzaki	2016	63	M	Endocarditis 10 years ago	No	AVR (bio 21 mm) AscAO	Centrifuge	-	Moderate hypothermia	Cold, antegrade and retrograde (Frem’s)	182	234	Minimal	RBC	Haptoglobin	18	No	No
21	Huenges	2017	15	F	Premature birth	-	HVAD, HeartWare (transplant 6 weeks after)	-	-	-	-	-	-	-	NO	-	-	RV failure, prolonged mechanical ventilation. Death 2 weeks after HTX	No
22	Menon	2020	7 months	F	Down syndrome	No	VSD closure MVpl ASD closure	Roller pump	Packed RBCs, albumin	Mild hypothermia	-	89	121	-	RBC, FFP	Ultrafiltration	6	No	No
23	Stevens	2021	19 months	F	-	No	AVSD closure	Roller pump	Exchange transfusion of RBCs and FFP	mild hypothermia	Cold, antegrade (del Nido)	35	57	-	RBC, FFP, PLT	Exchange transfusion	4	No	No

*AS* atherosclerosis, *ASD* autism spectrum disorder, *AVR* aortic valve replacement, *AVSD* atrioventricular septal defect, *Bio* bioprosthesis, *CABG* coronary artery bypass graft surgery, *CM* cardiac malformation, *CNS* central nervous system, *CPB* cardiopulmonary bypass, *CVA* cerebrovascular accident, *DCM* dilated cardiomyopathy, *DL* dyslipidemia, *F* female, *FFP* fresh-frozen plasma, *HF* heart failure, *HSM* hepatosplenomegaly, *HT* arterial hypertension, *HTX* heart transplantation, *HVAD* HeartWare ventricular assist device, *IABP* intra-aortic balloon pump, *IHD* ischemic heart disease, *LA* left atrium, *LOS* length of hospital stay, *M* male, *Mech* mechanical prothesis, *MVpl* mitral valve valvuloplasty, *MVR* mitral valve replacement, *OPCAB* off-pump coronary artery bypass, *PDA* patent ductus arteriosus, *PLT* platelets, *PT* pulmonary trunk, *PV* pulmonic valve, *RBC* red blood cells, *rHuEPO* recombinant human erythropoietin, *RV* right ventricle, *TVR* tricuspid valve replacement, *VSD* ventricular septal defect

**Table 3:** Perioperative laboratory tests

				**Hemoglobin (g/dl)**		**Hematocrit (%)**		**Plasma free hemoglobin (mg/dl)**		**Total bilirubin (mg/dl)**		**Haptoglobin (mg/dl)**		**Reticulocytes (%)**		**Creatinine (mg/dl)**	
	**Author**	**Year**	**Age**	**Preop**	**Postop (min–max)**	**Preop**	**Postop (min–max)**	**Preop**	**Postop (min–max)**	**Preop**	**Postop (min–max)**	**Preop**	**Postop (min–max)**	**Preop**	**Postop (min–max)**	**Preop**	**Postop (min–max)**
1	Moyes	1971	16	13	-	-	-	24	(36)	0.60%	-	-	-	0.5	-	-	-
2	Moyes	1974	5	-	-	-	-	12	(18–45)	-	-	-	-	-	-	-	-
3	deLeval	1974	x	13.5	(13.5)	-	-	-	(< 40)	-	-	-	-		-	-	-
4	Chun	1982	38	15.4	-	43	-	-	-	-	-	-	-	5	-	-	-
5	Gayyed	1993	67	12.1	(7.5–10.7)	-	-	-	-	5.2^a^	(2.6)^a^	-	-	40	(8)	-	-
6	Gayyed	1993	60	13.1	(12.1)	-	-	-	-	4.5^a^	(6.0)^a^	-	-	12	(5)	-	-
7	Gayyed	1993	64	11.3	(10)	-	-	-	-	-	(4.2)^a^	-	-	1	(1)	-	-
8	Aykac	1994	15	-	-	-	-	-	-	-	-	-	-	-	-	-	-
9	Kawahira	1994	15 months	9.6	-	-	-	-	-	1.5	(2.2)	< 2.5	(4.6)	47	-	-	-
10	Dal	1995	31	12.5	-	-	-	6	(8–10)	-	-	-	-	0.5%	(2%)	-	-
11	Yamagishi	1998	50	9.8	-	-	-	-	-	3.6	-	-	-	-	-	-	-
12	Aoyagi	2001	9	14.2	-	-	-	6,8	(12.9–44.9)	0.77%	-	-	-	10.10	-	-	-
13	Walther	2005	6 weeks	-	-	-	-	-	-	-	-	-	-	-	-	-	-
14	Yoshimura	2006	18 months	7.2	(14.1–16.4)	-	-	-	-	2.8	(2.9–4.1)	-	-	36.8	-	-	-
15	Johnson	2010	6	8.7	(12.5–15.7)	24.8	(37–46)	-	-	-	-	-	-	-	-	0,5	-
16	Khatri	2010	61	13.2	-	-	(26)	-	-	0.6	-	-	-	-	-	-	-
17	Imantalab	2011	60	13.9	(5–10)	39.9	-	-	-	1	-	-	-	-	-	-	-
18	Spegar	2015	69	-	-	-	-	130.5	(98.6)	1^a^	(1.3–3.1)^a^	-	-	-	-	0.92^b^	(0.86–1.02)^b^
19	Hargrave	2015	28	-	-	27	(18–29)	-	-	3.9	(3.6)	-	(< 20)	12.8	(15.7–22.2)	-	-
20	Matsuzaki	2016	63	8.7	(7.9–10.5)	23.8	(24.7–29.8)	-	-	3.3	(6.6)	24	(< 10)	-	-	0,93	-
21	Huenges	2017	15	-	-	-	-	-	-	-	-	-	-	-	-	-	-
22	Menon	2020	7 months	11.8	(10.4–14.1)	35	(31–44)	-	-	4.1	(2.7–2.8)	-	-	3	(4.1)	-	-
1	Stevens	2021	19 months	10	(12)	30.6	(37.7)	10	(70)	2.1	(2.1)	< 1	(27)	-	-	0.25	(0.23)

^a^Serum bilirubin concentration was converted from µmol/l to mg/dl by dividing by 16.81 (Wang et al. 2023)

^b^Serum creatinine concentration was converted from µmol/l to mg/dl by dividing by 88.4 (Paige and Nagami 2009)

## Case study

A 38-year-old patient was admitted to the Department of Cardiac Surgery, Upper-Silesian Medical Centre in Katowice, Poland, in mid-2022 for aortic valve surgery. His medical history included HS, splenectomy (2012), gallbladder stones, diabetes mellitus, history of Covid-19, and several previous hospitalizations for infections. The calculated EuroSCORE was 1.08%. The transthoracic echocardiography left ventricular ejection fraction was 50%, and the aortic valve was bicuspid with severe regurgitation. No other valvular defects or ascending aorta dilatation were noted. Preoperative laboratory tests showed no significant abnormalities with reticulocytes count 1.6%.

After preparation, the surgery was performed. Intraoperatively, a bicuspid valve type 1 with a 30-mm aortic annulus was visualized. Annuloplasty was performed with a GORE-TEX Suture narrowing the ring to 23 mm, then raphe was decalcified, and the right to left cleft was closed, resulting in equal leaflets and a coaptation of 9 mm. Total cardiopulmonary bypass time was 38 min, with 33 min of aortic cross-clamping. During the surgery, Sorin Biopump, Euroset drains (aortic cannula 22 mm, common venous cannula 32 mm), Inspire oxygenator, and cold Del Nido cardioplegia were used. Standardized cardiac output calculated to body surface area was maintained intraoperatively, and norepinephrine infusion was administered with a maximum dose of 0.015 µg/kg/min. The lowest core temperature recorded was 35.5 °C. The lowest hemoglobin value was 11.2 g/l, hematocrit 33%, and the maximum lactate concentration 1.5 mmol/l. No blood or blood products were transfused. Total intraoperative diuresis was 200 ml of clear urine. Follow-up transesophageal echocardiography showed a trivial jet of aortic regurgitation with no hemodynamic significance.

The postoperative course was negligible; he was discharged home on day 6. During 3 days in the intensive care unit (ICU), total drainage was 660 ml (maintained 32 h), total diuresis stimulated by a single dose of diuretic was 4850 ml, and 2880 ml of crystalloids was administered. No blood or blood components were given. The patient was hemodynamically stable with a total norepinephrine infusion time of 16 h and a maximum dose of 0.15 µg/kg/min. No clinical or biochemical features of hemolysis were observed. Laboratory results are shown in Table 4. Written consent was obtained from the patient to describe the case.

**Table 4:** Results of the patient’s preoperative and postoperative laboratory tests

		Before surgery	Day 0	Day 1	Day 2	Day 3
Hemoglobin	g/dl	17.4	13.2	12.0	11.2	11.4
Hematocrit	%	50.1	36.0	33.5	31.9	31.3
Platelets	G/µl	437	236	278	243	288
Creatinine	mg/dl	-	-	0.60	0.64	0.61
uACR	mg/g	-	-	55	< 30	< 30
ALT	U/l	36	-	20	16	20
AST	U/l	27	-	36	55	25
ALP	U/l	-	-	-	54	58
GGTP	U/l	-	-	6.3	7.7	9.2
LDH	U/l	-	-	247	-	250
BR total	mg/dl	1.0	-	1.2	1.1	0.5
BR direct	mg/dl	-	-	0.58	0.3	0.22
BR indirect	mg/dl	-	-	0.58	0.28	0.13
hsTn	ng/ml	-	-	0.148	-	-
pfHb	mg/dl	-	8.9	8.6	9.6	-

*ALP* alkaline phosphatase, *ALT* alanine aminotransferase, *AST* aspartate aminotransferase, *BR* bilirubin, *pfHb* free hemoglobin, *GGTP* gamma-glutamyl transferase, *hsTn* high sensitivity troponin, *LDH* lactate dehydrogenase, *uACR* urine albumin/creatinine ratio

## Discussion

Patients suffering from HS are potentially at the high risk of perioperative complications as a result of hemolysis. There are currently two approaches to patient treatment: strict control of hemolysis triggers, intending to lower the risk of hemolytic breakthrough, and a liberal approach, with no deviation from the standard of care for patients undergoing cardiac surgery. There is still no study comparing these different strategies, and evaluating these approaches is further hindered by the disorder’s rarity and the variety of HS symptoms among patients. In our systematic review of the literature, the incidence of hemolysis among patients with congenital spherocytosis was 21.7% and did not seem to be affected by the technical aspects of the procedure. Similarly, the presented case indicates that the standard approach to cardiac surgical techniques and perioperative care did not increase the risk of early complications in patient with mild/moderate HS. Nevertheless, managing the HS patient requires knowledge of the disease and potential risk factors for hemolysis.

When red blood cells are destroyed, the hemoglobin is released into the bloodstream. This can lead to several different outcomes. Free hemoglobin binds to haptoglobin, a protein that limits hemoglobin loss through the urine and facilitates hemoglobin uptake in the reticuloendothelial system. The breakdown of hemoglobin in the reticuloendothelial system releases the heme moiety, which is then converted into biliverdin and bilirubin. The accumulation of bilirubin can result in hyperbilirubinemia and jaundice. Moreover, if the hemolysis rate is high, the haptoglobin may be depleted, and free hemoglobin can accumulate in the bloodstream, resulting in severe cellular and organ damage.

The heme moiety is a potent source of reactive oxygen species, which can cause cellular damage and inflammation. Direct effects of free hemoglobin include injury to organs and vascular endothelium, which can result in endothelial dysfunction and impair the proper microvascular function. Furthermore, free hemoglobin limits NO bioavailability, thereby contributing to vasoconstriction and impaired organ blood flow (Reiter et al. 2002). These possible effects can be particularly pronounced in people with preexisting cardiovascular diseases such as hypertension, atherosclerosis, or heart failure.

Besides cardiovascular impact, increased free hemoglobin in the bloodstream can cause organ damage. The kidneys are highly susceptible, while they filter and excrete excess hemoglobin. The accumulation of heme-containing proteins in the kidneys causes direct cytotoxicity, intrarenal vasoconstriction, and intratubular cast formation, which can cause acute kidney injury or exacerbate chronic kidney disease (Khalighi et al. 2015).

Red blood cells elongate under strong external forces without rupturing and tend to return to their previous shape after the force ceases, unless they have a structural defect as in the HS. During surgery, it is crucial to remember that preventing hemolysis requires the collaborative efforts of the surgeon, perfusionist, anesthesiologist, and the nursing staff. To ensure good understanding of this issue, it is important to highlight several aspects of perioperative care.

### Cardiopulmonary bypass

#### The pump

Of the different types of cardiac surgery, the most common are those involving CPB, which, due to their construction, are potentially the main source of hemolysis. Red blood cells are irreversibly damaged during CPB, but hemolysis is usually minimal in patients without RBC disorders when CPB is properly managed (Vercaemst 2008). However, data from one study indicate that up to 15% of the RBC subjected to the extracorporeal circulation may be irreversibly damaged, and removed during the 24-h period (Valeri et al. 2006). When blood is pumped through the external circuit, it is exposed to physical and mechanical stress. The main component of the extracorporeal circulation system is the pump which design and mechanism of action depend on the manufacturer. Currently, there are two main types: nonocclusive roller pumps and centrifugal pumps. Old-generation CPB with occlusive rollers could potentially induce hemolysis, although an in vitro experiment showed no significant hemolysis in the blood of a HS patient during an occlusive CBP pump (Moyes et al. 1971). The modern CPB with both roller and centrifugal do not differ in hemolysis in healthy population (Saczkowski et al. 2012). The most recent meta-analysis showed higher pfHb concentrations with the use of roller pumps; however, there were significant heterogeneity and inconsistency — the authors conclude that centrifugal pump may be superior to roller pump in terms of pfHb levels but only when the duration of CPB exceeds 90 min (Bhirowo et al. 2023). During extracorporeal circulation in some types of roller pumps, the perfusionist can impose laminar or pulsatile flow. Pulsatile flow can potentially generate a higher circulatory pressure gradient and, thus, higher shear forces than non-pulsatile flow. It has been suggested that using pulsatile flow leads to increased concentration of free hemoglobin and blood cells depletion. However, experimental study suggest that pulsatile perfusion has no significant difference from non-pulsatile perfusion in terms of hemolysis and deformability of RBCs (Kang et al. 2010). Conclusions from a recent meta-analysis are similar — pulsatile flow may potentially increase the incidence of hemolysis, but the literature data are inconsistent (Bhirowo et al. 2023).

#### The cannulas and oxygenator

In a cannula, the shear stress depends on the radius, length, and the pressure gradient across the cannula or oxygenator. Most oxygenators’ shear stress levels are substantially below hemolysis or even sublethal RBC damage thresholds (De Somer et al. 1996). Also, meta-analysis by Bhirowo et al. did not found significant differences between two most common types of oxygenators — membrane and hollow-fiber membrane oxygenator (Bhirowo et al. 2023).

As blood flows at high speed through a small cannula, shear stresses act on red blood cells, resulting in a change in their shape. In addition, small cannulas can cause turbulence in blood flow contributing to hemolysis. Wider cannulas are associated with less erythrocyte injury. Compared to curved tips, hydrophobic cannulas with straight tips result in less turbulent flow and potentially (not certain) less hemolysis. A pressure gradient below 100 mmHg is considered safe (Burt et al. 2015). Using biocompatible materials and maintaining appropriate anticoagulation can prevent blood clots’ formation and reduce the risk of turbulent flow. Heparin-coated polyvinyl chloride PVC tubing can cause some hemolysis when compared to other materials, but it is attributed to the ex vivo cytotoxicity of the material components, which can occur during prolonged blood contact in the tube during laboratory tests, rather than hemolysis during clinical use. The degree of hemolysis is relatively low (about 1%); hence, the material cannot be considered hemolytic (Harmand and Briquet 1999).

#### Priming

Priming is often debated regarding hemodilution and thus hemolysis. While numerous publications highlight the possible decrease in hemoglobin concentration and thus potential reduction in global oxygen delivery (DO2) during massive transfusions of crystalloids and colloids, the essence of the problem of hemodilution anemia is the decrease in Hb concentration rather than the absolute value. Potentially, a decrease in the concentration of other plasma constituents including proteins and electrolytes can induce abnormal oncotic pressure resulting in hemolysis. An in vitro study found that hemodilution of RBCs with a large volume of phosphate-buffered saline (PBS) considerably increases RBC shear stress susceptibility when compared to RBCs diluted with plasma. Plasma’s protective effects can be attributed to the fact that albumin and other negatively charged plasma proteins bind to the surface of cells, drawing sodium ions within. This osmotically active layer may prevent RBCs from absorbing an excessive amount of water (Butler et al. 1992; Sargent et al. 2021). Sometimes, retrograde autologous priming is suggested to minimize the use of crystalloids.

#### Air-blood boundary and suction

There is a widespread belief that contact between blood and air in the venous reservoir, oxygenator, left ventricle venting, and suction can cause hemolysis during CPB. This belief may only be partially true. In an in vitro model, it has been shown that hemolysis is induced not by simple exposure to air alone but by a combination of air exposure and negative pressure. The amount of hemolysis is directly related to the vacuum applied at the air-blood interface (Pohlmann et al. 2009).

This could be explained by the phenomena of cavitation, which is the rapid phase transition from the liquid phase to the gas phase at reduced pressure. The phenomenon is unstable, with gas bubbles constantly forming, collapsing, and reforming. The violent implosion of a bubble creates a sudden impact on the surrounding liquid, resulting in a strong local impulse pressure wave that strikes every boundary it encounters. When the bubble and its collapse are asymmetrical, a very high velocity (up to 300 m/s) liquid microburst is formed, which causes damage when it strikes the cellular membrane (Wielogorski et al. 1975). Also, surface tension of air bubbles can cause red blood cells to rupture as they move through narrow blood capillaries (Bento et al. 2018).

Nonetheless, producers develop various CPB circuits to minimize blood-air contact. Using closed-circuit CPB (such as the Jostra MECC System) may potentially minimize hemolysis, activation of the coagulation cascade, and inflammatory response because of the absence of a venous reservoir and thus minimal blood-air contact. Also, the system’s small volume decreases the risk of hemodilution. However, because it lacks a venous reservoir, its application seems to be restricted to low-bleeding risk procedures (Fromes 2002).

Cardiotomy suction is one of the main causes of hemolysis, also contributing to the systemic inflammatory response by activating leukocytes, platelets, and the complement system (Skrabal et al. 2006). Hemolysis appears to be insignificant during roller pump assist cardiotomy suction (RPA), but system occlusion causes a considerable increase in fHb. The critical pressure for blood morphotic element damage ranges from − 120 to − 600 mmHg. When the suction tip is obstructed, the inlet pressure can exceed − 600 mmHg, resulting in cavitation, turbulent flow, and significant shear stress (Jahren et al. 2021; Vercaemst 2008).

There are several solutions to the suction occlusion issues. SmartSuction system consists of an optical sensor on the tip that sucks blood only when the sensor detects blood, minimizing the mixing of blood and air. The SmartSuction operates between − 20 and − 150 mmHg, depending on the blood-air ratio of the suction cannula, and produces a suction flow of 0.5–4.0 l/min. This device has demonstrated less hemolysis, although literature data is not consistent (Jegger et al. 2007; Michinaga et al. 2019; Stalder et al. 2007).

Potentially, suction exposes blood to air during passage through the drain and can greatly accelerate hemolysis due to the differing viscosities of blood and air. It has been demonstrated that developing new suction methods, including those that are fluid-driven using the Venturi effect, can lower the degree of hemolysis by limiting the contact area between blood and air (Arensdorf et al. 2018).

Also, most commonly used CPB systems have the cardiotomy reservoir integrated with the venous reservoir, and the filtered blood (20–40 µm) is mixed with the venous blood. There are so-called two-headed (Dihead) CPB circuit systems in which it is possible to separate the two reservoirs, making it possible to flush the extracted blood and transfuse it to the patient. Spherocytes appear to be resistant to the process of washing and centrifugation with the use of red blood cell washing devices (such as CellSaver). This results in a reduction of reinfused fHb (Pierangeli et al. 2001).

#### Cardioplegic solution

Cold cardioplegia can induce hemolysis, while RBC membranes become more rigid in cold temperatures, limiting the cell’s capacity to adapt and pass through narrow blood capillaries. RBCs are also more vulnerable to mechanical and shear stress, while cold temperatures can alter the membrane and cytoskeleton of RBCs (Takahashi et al. 1986). Aside from these physical changes, exposure to low temperatures can activate the immune system, damaging morphotic elements of the blood (Swiecicki et al. 2013). When administering normothermic cardioplegia, larger volumes and more frequent applications may be required to achieve and maintain the desired effect. Larger volumes may increase the risk of local hemodilution. In the literature analysis, the use of cold cardioplegia was described in six patients; one patient was diagnosed with hemolysis (Imantalab et al. 2011). In other cases, the temperature of the cardioplegia solution was not specified. Given the fact that the most commonly used solution in clinical practice is cold cardioplegia, there is a possibility that this type of cardioplegia was also used in other cases, and thus, it should be analyzed with caution.

#### Ultrafiltration

Hemoadsorption (HA) of cytokines using special filters such as CytoSorb has the potential to reduce inflammatory responses and improve patient outcomes. CytoSorb is used in cardiac surgery, but literature data have not shown a significant reduction in pro-inflammatory cytokine concentrations during CPB while using CytoSorb. Only longer-lasting concentrations of the anti-inflammatory IL-10 have been observed, with no clinically significant effect on the course of treatment (Bernardi et al. 2019; Holmén et al. 2022; Taleska Stupica et al. 2020). CytoSorb can potentially bind free hemoglobin, but there was no significant decrease in free hemoglobin in patients treated with hemadsorption. A decrease in haptoglobin levels on the first postoperative day in patients who did not receive hemadsorption was shown, which may indirectly indicate some adsorbing effect on free hemoglobin (Bernardi et al. 2016). Also, ultrafiltration reduces hemodilution. In the systematic review, the use of renal replacement therapy (RRT) has been described in four patients, only one with hemolysis (Imantalab et al. 2011).

### Surgical management

#### Procedure time

A study of 22 patients undergoing CABG showed that more than one-quarter of total hemolysis occurs in the first 5 min of extracorporeal circulation (Vieira Junior et al. 2012). Also, in a study by Passaroni et al., there was a significant within-group difference in haptoglobin concentration between the pre- and post-CPB periods in different pump types, which suggest that the duration of CPB may an important factor of hemolysis (Passaroni et al. 2018). There is no clear data in the literature to indicate that prolonged surgery time or CPB time affects the degree of hemolysis, and a threshold time after which the risk is higher has not been established. An experimental study on the extracorporeal membrane oxygenation (ECMO) system showed that the increase in pfHb became significant at 240 min but only with circulating heparinized blood. Regardless of ECMO flow, pfHb was not observed in citrated blood over time. Interestingly, an ex vitro study showed that the degree of hemolysis was higher in male blood donors (Chan et al. 2021). Organ damage, particularly acute kidney injury, observed in clinical practice during prolonged procedures can be caused by various of causes, including but not limited to pfHb.

#### Bioprosthesis vs mechanical prosthesis

Cardiac prosthesis-related hemolytic anemia (CPHA) is still a subject of research. An older generation mechanical prosthesis has a potentially higher risk of subclinical intravascular hemolysis, which may have been more than 50% in St. Jude Medical (SJM) bileaflet design valves implanted in the late 1980s and early 1990s (Skoularigis et al. 1993). Among the new valves, the incidence of mild subclinical hemolysis has decreased significantly but may still be 26% in patients with mechanical prostheses (CarboMedics, Sorin Bicarbon) and 5% with bioprostheses (St. Jude Medical Toronto, Baxter Perimount, and Medtronic Mosaic). Also, the position of implantation may be important — mitral valve replacement versus aortic valve replacement were correlated with the presence of hemolysis (Mecozzi et al. 2002). It is widely believed that paravalvular leak (PVL) is a predisposing factor for hemolysis. However, it should be noted that PVL is relatively common and occurs in approximately 6% of patients with aortic valve prosthesis and 32% of patients with mitral valve prosthesis at the time of discharge after surgery. Although lactate dehydrogenase concentration was higher in patients with paraprosthetic jets than in those without, hemoglobin and haptoglobin concentrations were not different which suggests clinically insignificant hemolysis (Ionescu 2003). Also, subclinical hemolysis after On-X aortic valve mechanical prothesis occurs in 14.3%, but the proposed mechanism involves rather patient-prosthesis mismatch, rather than the degree of PVL, reaching 33.3% for the smallest protheses (Perek et al. 2017). In our literature analysis, mechanical valves were implanted in three patients; none was diagnosed with hemolysis (Chun et al. 1983; deLeval 1974; Yamagishi et al. 1998).

#### Valve repair

Hemolytic anemia is not common after valve repair. In general population of 1095 patients who required reoperation after mitral valve repair, only 10 was reoperated for hemolytic anemia reason (Cerfolio 1997). Similar to artificial valve hemolysis, mechanisms may include jet collision with the prosthetic ring, jet fragmentation by a dehisced annuloplasty ring, and jet acceleration through a para-ring channel (Ward et al. 2000). In the systematic review, two of five patients undergoing valvular repair were diagnosed with hemolysis (Gayyed et al. 1993; Hargrave et al. 2016).

#### Preemptive splenectomy

Considering the risks outweighing the potential benefits, current guidelines recommend splenectomy only in patients with severe HS (*Hb* < 8 g/dl, reticulocyte count > 10%, and bilirubin > 51 µmol/l) and only for those over 6 years of age. Additional indications for splenectomy in intermediate categories are factors affecting quality of life such as symptomatic/painful splenomegaly with thrombocytopenia or leukopenia (Iolascon et al. 2017). In the general population undergoing splenectomy, an increased risk of overwhelming post-splenectomy infections (OPSI) and more than fivefold higher incidence of stroke, myocardial infarction, and coronary or carotid artery surgery were observed (Schilling 1997). Ten of 23 patients analyzed in the systematic review underwent splenectomy prior cardiac surgery.

### Laboratory tests

Among the numerous biochemical tests, the classic diagnosis of hemolysis is based on the assessment of reticulocytosis, elevated lactate dehydrogenase, elevated unconjugated bilirubin, decreased haptoglobin, and hemoglobinuria. However, it is important to remember that LDH is an intracellular enzyme and a nonspecific marker, as it can be elevated in many situations, including myocardial infarction, liver dysfunction, hemolysis, myopathies, and other (Herman et al. 2019). Plasma-free hemoglobin (pfHb) is a direct marker of hemolysis, but its quantification is not always available. Recently, Shin et al. proposed a device for point-of-care testing of plasma-free hemoglobin and hematocrit for mechanical circulatory support using colorimetry (Shin et al. 2021).

### Treatment

Blood product transfusions to compensate for oxygen carrier loss, administration of vasoactive drugs, or end-organ damage protection are crucial for patients with intravascular hemolysis. Patients with heart failure and coronary disease may not tolerate anemia due to their reduced ability to improve cardiac output. This leads to a decrease in DO2 and organ hypoxia, which in the case of the heart cannot be fully compensated by increased oxygen extraction, leading further to myocardial ischemia. Therefore, the recommended hemoglobin transfusion threshold is 7 g/dl for stable critically ill patients (including patients with moderate ischemic heart disease), with a target value of 7–9 g/dl. In patients with acute coronary syndrome, higher threshold values of 8.5–9.5 g/dl may be appropriate improving long‐term cardiovascular outcomes (Hébert et al. 1999; Mistry et al. 2023).

#### Haptoglobin

In addition to the free hemoglobin, bilirubin, LDH, and reticulocyte count, haptoglobin is a parameter that may indicate the presence of intravascular hemolysis. Haptoglobin is an acute-phase protein that binds irreversibly to fHb preventing its glomerular filtration, and the complex is phagocytized in the mononuclear phagocyte system. The half-life of free haptoglobin is approximately 5 days whereas the hemoglobin-haptoglobin complexes just few minutes (Shih et al. 2014). Haptoglobin is common but not ideal marker of hemolysis. In the absence of hemolysis, reduced haptoglobin concentrations can be found in liver cirrhosis, disseminated ovarian cancer, pulmonary sarcoidosis, and some endocrinology diseases. Haptoglobin has already been used in cardiac surgery and may be a potent drug used in patients with hemolytic anemias (Tanaka et al. 1991). In our systematic review, haptoglobin was used in four patients but only one with intravascular hemolysis (Aoyagi et al. 2001; Kawahira et al. 1994; Matsuzaki et al. 2016; Yoshimura et al. 2006).

#### Vasodilatators and nitrous oxide

During intravascular hemolysis, reactivity of free plasma hemoglobin with nitrous oxide (NO) may affect vascular tone leading to vasoconstriction and impaired organ perfusion. Vasodilators such as sodium nitroprusside (which is a NO donor) may be beneficial in these patients. Also, inhaled NO oxidized most of the cell-free hemoglobin in the plasma to methemoglobin, blocking its endogenous NO-scavenging properties and therefore reduces vasoconstriction (Minneci 2005). In the study of patients undergoing multiple valve replacement, nitric oxide decreased the incidence of kidney injury (Lei et al. 2018).

#### Angeli’s salt—nitroxyl

Angeli’s salt (sodium alpha-oxyhyponitrite, Na2N2O3) is a nitroxyl anion donor, which reacts with cell-free oxyHb to form metHb, thereby reducing pulmonary and systemic vasoconstriction. Several animal studies are being conducted in various areas of medicine using nitroxyl delivered by Angeli’s salt (He et al. 2008).

#### Pentoxifylline

Pentoxifylline (PTX) is a methylxanthine increasing erythrocyte flexibility and reducing whole blood viscosity. Some studies suggest that PTX may be an effective agent in the treatment of hemolysis during CPB but also in patients with prosthetic heart valves and continuous-flow left ventricular assist device support (Golbasi et al. 2003; Jennings et al. 2013).

#### Polyethylene glycol

Polyethylene glycol (PEG) is a water-soluble linear polymer used, among other things, in the pharmaceutical industry as a component of artificial blood substitutes, a biocompatible drug delivery carrier, and as coating for biomaterials. Experimental studies on bovine blood showed that hemolysis of red blood cells suspended in plasma and PEG solutions was several times lower than in dextran and phosphate-buffered saline (PBS) solutions. PEG 20,000 was shown to produce a significant protective effect at concentrations as low as 0.1 g/ml. The proposed protective mechanism of PEG may be a result of coating the surface of the artificial material as well as the absorption of PEG into the RBC membrane complex, thereby increasing the resistance of RBCs to shear and stress (Kameneva et al. 2003).

#### Poloxamer 188

Purified poloxamer 188 (P188) is a linear copolymer with hemorheological properties improving microvascular blood flow. It may increase RBC membrane hydration, cause fibrinogen dispersion, and have anti-adhesive effects facilitating blood flow and reducing cell-fibrin/fibrinogen interactions in a many hemorheological disorders (Hoppensteadt et al. 2013). The use of poloxamer 188 was identified in one patient (Kawahira et al. 1994).

#### Sodium bicarbonate

In addition to IV fluid administration (which can be detrimental in patients with heart failure), a protective effect of sodium bicarbonate on the renal injury after cardiac surgery has been reported. The use of sodium bicarbonate, leading to urinary alkalization, may aid in the prevention of hemoglobin-associated pigment nephropathy, but there is no conclusive clinical evidence that an alkaline diuresis is superior than a saline diuresis in preventing acute kidney injury (Bailey et al. 2015; Haase et al. 2007; Huerta-Alardín et al. 2005). In a study by Wetz et al., sodium bicarbonate administration was beneficial to cardiac surgical patients with a baseline low risk of acute renal failure after cardiac surgery (Thakar score 3 < pts.), with no significant difference in the high-risk patient group (Wetz et al. 2015).

## Limitations

Our study has several limitations. A rare case of congenital RBC defect undergoing cardiac surgery was described, and extrapolating the results to a larger population may be flawed. Because of the rarity of the disease, the analysis of the literature concerns only single case reports from different decades. Comparisons between these publications may therefore not be appropriate. In addition, the quality of the reported data is poor, and it was not possible to perform an in-depth statistical analysis.

## Conclusions

The literature data show that hemolysis is not common in patients with HS undergoing various cardiac surgery techniques. The typical management of a patient with mild/moderate HS does not appear to increase the risk of significant clinical hemolysis. Commonly accepted beliefs about factors inducing hemolysis during cardiac surgery may not be fully justified and require further investigation.

## Data Availability

All data generated or analyzed during this study are included in this published article.

## Abbreviations

ALP: Alkaline phosphatase
ALT: Alanine aminotransferase
AS: Atherosclerosis
ASD: Autism spectrum disorder
AST: Aspartate aminotransferase
AVR: Aortic valve replacement
AVSD: Atrioventricular septal defect
Bio: Bioprosthesis
BR: Bilirubin
CABG: Coronary artery bypass graft surgery
CM: Cardiac malformation
CNS: Central nervous system
CPB: Cardiopulmonary bypass
CVA: Cerebrovascular accident
DCM: Dilated cardiomyopathy
DL: Dyslipidemia
DO2: Global oxygen delivery
ECMO: Extracorporeal membrane oxygenation
F: Female
FFP: Fresh-frozen plasma
GGTP: Gamma-glutamyl transferase
HF: Heart failure
HSM: Hepatosplenomegaly
hsTn: High sensitivity troponin
HT: Arterial hypertension
HTX: Heart transplantation
HVAD: HeartWare ventricular assist device
IABP: Intra-aortic balloon pump
IHD: Ischemic heart disease
LA: Left atrium
LDH: Lactate dehydrogenase
LOS: Length of hospital stay
M: Male
Mech: Mechanical prothesis
MVpl: Mitral valve valvuloplasty
MVR: Mitral valve replacement
OPCAB: Off-pump coronary artery bypass
PCV: Polyvinyl chloride
PDA: Patent ductus arteriosus
pfHb: Free hemoglobin
PLT: Platelets
PT: Pulmonary trunk
PV: Pulmonic valve
PVL: Paravalvular leak
RBC: Red blood cells
rHuEPO: Recombinant human erythropoietin
RRT: Renal replacement therapy
RV: Right ventricle
TVR: Tricuspid valve replacement
uACR: Urine albumin/creatinine ratio
VSD: Ventricular septal defect
